# HPV Testing, Self-Collection, and Vaccination: A Comprehensive Approach to Cervical Cancer Prevention

**DOI:** 10.3390/curroncol32110594

**Published:** 2025-10-23

**Authors:** Shannon Salvador

**Affiliations:** Jewish General Hospital, McGill University, Montreal, QC H3T 1E2, Canada; shannon.salvador@mcgill.ca

**Keywords:** HPV, vaccination, cervical cancer

## Abstract

This white paper, prepared by a consortium of Canadian national and provincial organizations and experts, outlines urgent strategies to curb the rising incidence of HPV-related cancers, of which, cervical cancer is currently the fastest-growing cancer in Canada. Despite school-based vaccination programs, the national HPV vaccine uptake remains suboptimal at about 64%, far below the 90% coverage target by 2025 necessary to eliminate cervical cancer by 2040. The report emphasizes a multi-pronged approach: support access to HPV vaccination with expanded funding policies and education around school-based programs while addressing inequities in underserved populations. HPV testing is highlighted as the preferred method for cervical cancer screening, offering higher sensitivity than Pap smears. Self-collection is presented as an innovative strategy to reduce barriers, particularly for marginalized groups, with promising evidence from Canadian pilots and international models. Crucially, we call for investment in comprehensive, population-based databases to track vaccination, screening participation, and follow-up care. Robust registries would allow targeted outreach to under- or never-screened individuals, ensure timely follow-up of abnormal results, and measure the impact of prevention programs across Canada. With vaccination, equitable access to HPV testing, integration of self-collection, and strong data systems, Canada can achieve its goal of eliminating cervical cancer within two decades.

## Simple Summary

In April 2025, advisors from across Canada, representing 10 provinces and 21 national or provincial organizations, met to discuss the importance of human papillomavirus (HPV) vaccination and screening in creating this report for the Canadian public and governmental agencies in healthcare.

## 1. Introduction

### 1.1. Key Messages: Public

***Did you know?***

Cervical cancer is the fastest growing cancer in Canada among women and other people with a cervix [1].

These cancers are primarily caused by infection with human papillomavirus (HPV).

Anyone can contract HPV through any skin-to-skin sexual contact, which includes genitals, skin, or mouth. Condoms do not fully protect people from this virus because there is still direct skin to skin contact.

The good news? Cervical cancers are preventable through vaccination and screening.

***How can I protect myself and my family?***

 **Get vaccinated against HPV**

- HPV vaccination is safe and effective in preventing most cancers caused by HPV in all sexes and genders [2,3,4].
- All provinces and territories offer HPV vaccines through school-based programs.
- You can also contract HPV vaccines at your pharmacy, primary care clinic, community clinic, or local walk-in clinic if you did not receive it in school.

 **Screening for cervical cancer saves lives**

- This can be performed with the HPV test or a PAP test. Ask your healthcare provider which is recommended for you. The HPV test is the preferred method for screening, and most provinces have a plan or have switched to primary screening with the HPV test.
- HPV self-collection may be available in your province or territory. This involves inserting a small swab in your vagina and can be performed in the comfort and privacy of your home or healthcare provider’s office.
- Women and other people with a cervix aged 25 and older should be screened regularly.

 **Follow up if you have a screening result that requires further evaluation**

- A healthcare provider will help you with the next steps.
- This usually requires a specialized exam called colposcopy.

You have choices. Choose cancer prevention.

HPV infection can cause nine different types of cancers: cervix, vulva, vagina, anus, penis, oral, throat, tonsils, and tongue [3,4,5]. There are three actions you can take to prevent cancer:

- Vaccinate your child now to protect them from cancer later.
- Participate in regular cervical screening to catch and treat any early changes before they become cancer.
- If you have HPV, follow up as guided by your healthcare provider.

### 1.2. Key Messages: Government

***Did you know?***

Cervical cancer is the fastest growing cancer in Canada [1].

Mouth and throat cancer are the second fastest growing cancers in Canada [1].

Both can be caused by HPV infection. HPV affects everyone.

HPV-related cancers are preventable.

***What can we do?***

Encourage HPV vaccination

- Support and advocate for school HPV vaccination programs to ensure all youths are vaccinated with ongoing education campaigns.
- Consider a policy in your jurisdiction, of “once eligible, always eligible” to allow publicly funded HPV vaccine access up to the age of 45.

Improve access to cervical screening

- Adopt HPV testing as the primary screening method for cervical cancer in all provinces and territories.
- Fund and expand HPV self-collection programs.
- Invest in HPV testing, a more effective and cost-efficient public policy solution.

Fund screening databases and follow up

- Promote comprehensive screening programs to include organized databases, communication, and recall reminders.
- Prioritize resources for high-risk, vulnerable, and marginalized population.
- Fund follow-up pathways for non-attached patients.

Canada set a goal to achieve 90% HPV vaccination coverage for all individuals by the time they reach 17 years of age by 2025. This would effectively eliminate cervical cancer by 2040 [6].

We are not there yet, but we can be.

Canadians deserve access to HPV vaccination as per national guidelines. HPV vaccination prevents cancers.

Canadians with a cervix deserve access to cervical cancer screening, with HPV testing, clinician- or self-collected.

Canadians who test positive for HPV must have access to timely and appropriate follow up and care.

We can prevent HPV-related cancers.

## 2. What Is HPV?

Human papillomaviruses (HPV) are DNA viruses. They infect the skin and mucosal soft tissue (tissue that lines body canals and organs in the digestive, respiratory, and reproductive systems) through direct contact [7].

Anyone can contract HPV.

HPV is common, and without vaccination, it is estimated that over 75% of people in Canada will be infected at least once during their lifetimes [8].

Because the initial HPV infection has no symptoms, most people do not know they have it [9].

Most people with a normal immune system will clear their HPV infections within two years, but the virus persists longer in about 20% of cases. The HPV infections that persist can lead to precancerous changes and eventually cancer. Progression to cancer can be slow, often requiring ten years or more after the original infection [1,10].

There are many genotypes of HPV, some much more serious than others. Persistent infection with high-risk HPV genotypes (e.g., 16, 18, 31, 33, 35, 39, 45, 51, 52, 56, 58, 59) causes various cancers like cervical, anal, vulvar, vaginal, penile, oropharyngeal.

Low-risk HPV genotypes (e.g., 6 and 11) usually do not cause cancer but do cause conditions such as anogenital warts (warts on the anus and genitals) and recurrent respiratory papillomatosis (wart-like growths around the voice box in the throat) [11].

## 3. How Is HPV Transmitted?

HPV is transmitted by skin-to-skin contact involving the genitals, including oral to genital contact, and nonsexual genital contact from mother to child during pregnancy or delivery [12,13,14].

Most people are exposed to HPV at some point in their lifetime [15].

## 4. Summary of the Current State of HPV in Canada

In April 2025, advisors from across Canada (see Appendix A), representing 10 provinces and 21 national or provincial organizations, met to discuss the importance of HPV vaccination and screening in creating this report.

### 4.1. HPV Vaccination

Canadian provinces and territories implemented school-based, publicly funded HPV immunization programs for girls between 2007 and 2010. By 2017–2018, all programs also included boys [16].

Canadian children are first eligible to receive HPV vaccines between the ages of 9 and 13, depending on the jurisdiction, and it is covered by provincial publicly funded vaccination programs until the completion of high school. Our immune systems are strongest when we are young; therefore, HPV vaccines work best when given around 9 to 13 years of age and before contact with the HPV virus. Some jurisdictions have extended financial coverage to adults at various age limits or include individuals at higher risk [6]. There are provinces (Manitoba, Prince Edward Island, and Newfoundland and Labrador) in Canada that have adopted a “once eligible, always eligible policy” to help reduce financial barriers to access if they did not receive the vaccine in grade school at the optimal time [6].

In 2020, Canada set a goal to achieve 90% HPV vaccination coverage of all 17-year-olds by 2025, and to eliminate cervical cancer by 2040 [6].

The most current Canadian data shows provincial/territorial HPV vaccination rates ranging from 47% to 81%, with an estimated national HPV vaccination completion rate of 64% in Canada [17].

### 4.2. Screening for Cervical Cancer

HPV causes more than 90% of cervical cancer, and cervical cancer is the fastest growing cancer in Canada [1].

Women and individuals with a cervix between the ages of 35 and 55 are the most likely to be diagnosed with cervical cancer [1]. This is also the age when people are likely contributing significantly to society, both socially and economically.

Screening needs to start 10 years before cervical cancer is typically diagnosed, when many HPV-positive individuals could be treated at the precancer stage. Thus, most screening programs start at the age of 25.

Percentage of eligible people screened for cervical cancer across Canada varies from 71 to 83% [16].

We need to do better. Screening with HPV testing is a better method of cervical cancer screening than the traditional Pap test alone. Despite the proven success of HPV screening, particularly for populations at highest risk of under-screening, its reach remains limited as it has been introduced in only four Canadian provinces thus far. Canada needs a comprehensive approach that includes multi-level interventions spanning patients, providers, and systemic levels to improve screening and reduce the incidence of cervical cancer in order to reach our goal of cervical cancer elimination.

## 5. HPV Vaccines in Canada

There are two HPV vaccines available in Canada. GARDASIL^®^9 (9vHPV) vaccine provides protection against infection caused by nine HPV types: 6, 11, 16, 18, 31, 33, 45, 52, and 58. CERVARIX^®^ (2vHPV) vaccine provides protection against infection caused by HPV types 16 and 18 [11]. The 9vHPV vaccine is the one used for the school-based vaccination programs.

The HPV vaccine is a preventative vaccine, so getting vaccinated will not alter the course of a current HPV infection [11]. However, even among individuals who have already been infected with one or more HPV types, the HPV vaccine will provide protection against the other HPV types contained in the vaccine, with the goal of preventing future HPV-related disease [11].

### 5.1. Vaccine Uptake

While most Canadian jurisdictions have publicly funded HPV vaccination programs for children between the ages of 9 and 13 (Table 1), vaccination uptake varies significantly by province and territory and within these jurisdictions. Very little data has been collected on the coverage of HPV vaccination for sub-populations within provinces and territories [18]. A review article from 2024 estimates that only 64% of Canada’s eligible population (who could have been vaccinated in the school-based programs) has been vaccinated against HPV [17]. This low HPV vaccination rate is likely not due to vaccination hesitancy, as data for completion of other available vaccinations (i.e., a COVID vaccination rate of 85% in the general population) suggests that the majority of Canadians strongly support vaccinations [17].

We know that the COVID pandemic had a significant negative impact on HPV vaccination across the country due to school closures. We also know that vaccination uptake is generally lower among males, people living in rural areas, smaller communities, northern communities, Indigenous people, people with lower socioeconomic status, and other at-risk populations [18,19].

**Table 1:** Provincial and territorial HPV vaccination school-based programs, current as of January 2025 [20].

Province/Territory	Grade HPV Vaccination Available
British Columbia	Grade 6, 2 doses
Alberta *	Grade 6, 2 doses
Saskatchewan	Grade 6, 2 doses
Manitoba	Grade 6, 2 doses
Ontario	Grade 7, 2 doses
Quebec *	Grade 4, 1 dose
New Brunswick	Grade 7, 2 doses
Nova Scotia	Grade 7, 2 doses
Prince Edward Island	Grade 6, 2 doses
Newfoundland and Labrador	Grade 6, 2 doses
Yukon	Grade 6, 1 dose
Northwest Territories	Grade 4, 5, or 6 (starting age 9 years), 2 doses
Nunavut	Grade 6, 2 dose

* Quebec and Alberta offer a catch-up vaccination program in secondary school.

### 5.2. HPV Vaccination Makes a Difference

HPV vaccination could prevent over 90% of cancers caused by HPV. HPV vaccination also prevents anogenital warts caused by HPV infection [2,7,21].

A meta-analysis [21] looked at data from 2014 to 2018 from 14 high-income countries, including Canada, with HPV vaccination programs in place for five to eight years. The study reported the following:

- 83% reduction in the two main cancer-causing HPV types (16 and 18) in girls aged 13 to 19 years, and 66% reduction in women aged 20 to 24 years.
- 67% decrease in anogenital wart diagnoses in girls aged 15 to 19 years, a 54% decrease in women aged 20 to 24, and a 31% decrease in women aged 25 to 29 years.
- The incidence of precancerous lesions was reduced by 51% in women who were vaccinated between the ages of 15 and 19, and by 31% in women who were vaccinated between the ages of 20 and 24 years.

A study from British Columbia [22] looked at the incidence of precancerous conditions caused by HPV that can progress into cervical cancer from 2004 to 2017 in women aged 16 to 23 years. Compared to women who were not vaccinated, they found a 62–65% reduction in various precancerous lesions in the vaccinated population. More recent data from the UK and Sweden show a significant reduction in the cervical cancer rate when HPV vaccination is given below that the age of 17, preferably at the ages of 12–13, with an 87% reduction in cervical cancer [23,24].

### 5.3. National HPV Vaccination Recommendations

Canada’s National Advisory Committee on Immunization (NACI) updated its HPV vaccine recommendations in July 2024 (Table 2) [25]. The Public Health Agency of Canada (PHAC) HPV vaccines: Canadian Immunization Guide is in agreement with these recommendations [11].

NACI and PHAC both recommend one dose of the HPV vaccine for individuals 9 to 20 years of age. Some jurisdictions, as indicated in Table 1, have already implemented 1-dose vaccination in school-based programs. Other jurisdictions may wait for additional data from ongoing trials before changing from a 2-dose to a 1-dose schedule. Studies that have assessed one-dose vs. two- or three-dose strategies have shown that a single dose of HPV vaccine proved highly effective in preventing persistent oncogenic HPV infection from HPV 16 and 18, the two strains responsible for the majority of HPV-related cancers, and will follow for long-term data about the efficacy of prevention of precancerous lesions [19,20,26].

## 6. Recommendations: HPV Vaccines

1. Support and advocate for school HPV vaccination programs to ensure all children are vaccinated.
2. Encourage catchup programs to ensure that every Canadian up to the age of 17 years receives at least one HPV vaccination dose.
3. Consider a policy and evaluate the benefits in your jurisdiction, of “once eligible, always eligible” to allow publicly funded access up to the age of 45.
4. Develop, implement, and evaluate targeted public education and community- and population-based strategies to identify and then address specific reasons for low vaccination rates with different populations.
5. Identify, develop, implement, and evaluate targeted strategies to address inequities resulting in reduced vaccine uptake in specific populations, e.g., remote and northern communities, Métis, Inuit and First Nations, new Canadians, and others.
6. Support provincial registries to more effectively identify unvaccinated individuals to allow for targeted education and encourage vaccination.
7. No other single tool is as effective in preventing HPV associated cancers as the HPV vaccine.

## 7. HPV Testing

### 7.1. HPV and Cervical Cancer

In 2016, there were 6.6 cervical cancer cases per 100,000 women in Canada. In 2023, the incidence was estimated to have reached eight cases per 100,000 women [1].

Cervical cancer is the fastest growing cancer in Canada [1].

This is a rising incidence of a disease that is almost completely preventable.

This increasing trend has been hypothesized to be associated with suboptimal screening uptake, including less intensive screening in more recent years, difficulty with accessing screening, and lack of follow up after screening [1].

Cervical cancer rates are especially high among rural and remote populations, and in people with low income. Smaller studies on marginalized populations indicate that rates are also likely to be higher among 2SLGBTQIA+ individuals and people of certain races or ethnicities, and very robust data shows a higher rate for people who identify as First Nations, Inuit, or Métis [27,28,29,30].

### 7.2. Other HPV-Related Cancers

The majority of HPV-related cancers (near two-thirds) occur beyond the cervix, and these cancers can affect both females and males (Table 3) [2,5,11,14,31].

In 2024, these cancers accounted for over USD 300 million in health system costs in Canada. Health system costs related to all aspects of cancer care are expected to increase over the next 10 years [32].

Despite progress in cancer research, rates of HPV-related cervical cancer, oral cancer, and throat cancer are increasing in Canada [1]. Oropharyngeal (mouth and throat) cancer is rising rapidly and is 4.5 times more prevalent in males than in females, emphasizing the need for all Canadians to be vaccinated in the school-based programs [3]. In Canadian males and females, the onset of oropharyngeal and anal cancers usually occurs between 60 and 70 years of age. Diagnoses of vaginal, vulvar, and penile cancers typically peak over the age of 70 [11].

### 7.3. HPV Testing for Cervical Cancer

HPV testing-based screening strategies are highly sensitive for the detection of precancers of the cervix. It has greater sensitivity to detect HSIL (cervical cancer precursor cells) and glandular lesions than traditional Pap tests [33]. Pap tests detect cellular abnormalities that might eventually develop into cancer, while HPV screening specifically detects the presence of HPV infection, responsible for over 90% of cervical cancer [34]. In Canada, Pap testing is recommended every three years, while HPV testing is recommended every five years.

At the time of the writing of this document, British Columbia, Prince Edward Island, and Ontario, and Quebec have shifted to HPV testing as the primary screening method for cervical cancer. Other provinces and territories are at different stages of planning and implementation of HPV testing.

### 7.4. Cervical Cancer Underscreening

There are marked similarities between populations with lower HPV vaccination uptake and those who are under screened for cervical cancer. The same health inequalities exist, e.g., socioeconomic factors, geographic location, access to healthcare, and cultural beliefs. Marginalized populations such as ethnic minorities, individuals with low incomes, immigrants, and rural and remote communities are typically at a higher risk of cervical cancer due to lower participation in screening programs [18,35].

Fear, embarrassment, lack of trust in the healthcare system, and previous healthcare-related or sexual trauma are also significant factors in underscreening. With appropriate support, such as the adaptation of HPV-based screening, which can include HPV self-collection (addressed in Section 7.5), we can help bridge these gaps.

### 7.5. HPV Self-Collection

HPV self-collection (self-sampling) is a tool that may help increase cervical cancer screening uptake across Canada [36]. It can reduce barriers to screening, particularly in marginalized populations. Benefits include convenience, privacy, ease of use, comfort, increased reach and coverage, and reduction in barriers such as distance to clinician, mobility, culture, and unattached patients. The simple HPV swab in the vagina can be self-administered either in a clinician’s office or at home through a mail-in program.

Prior to implementing an HPV self-collection program, the most critical aspect is laboratory validation with analytical agreement between self-collected and provider-collected sample types. Other elements include the development of patient and provider materials, strategies for user engagement, identifying a process to support unattached patients, developing/configuring an IT system for kit requests, mailing, and follow-up [37,38].

Self-collection is not simply the rollout of a new test. It is a process with a sequence of steps, and each step must be tested and function effectively. Success will depend on deliberate program design, targeted support, and a commitment to learning from real world data only by continuously monitoring and creatively assessing those outcomes in countries that have already introduced HPV self-collection. It will also require a coordinated and funded system for follow-up of abnormal results, particularly for patients unattached to a primary care provider.

### 7.6. British Columbia’s Self-Collection Experience

British Columbia ran a province-wide two-year pilot test from 2022 to 2023 to assess and optimize program elements for HPV self-testing in the province, which included an in-office and a mail-in self-collection option [36]. They identified the need for linked clinics to work with unattached patients who test positive for HPV and thus require follow up and cytology. They have found that it takes up to 40 weeks to obtain 75% of kits back, and that younger patients took longer to return kits. Older patients return up to 87% of kits, while younger patients only return 68%. Kits were optimized by using directive and empowering language, a social media campaign, and phone calls. Individuals who test positive for ‘HPV Other’ (high risk types other than HPV 16 and HPV 18) are directed to go to providers for follow up, but some people who choose self-collection are unattached and are at risk for loss to follow up. Ensuring unattached patients are connected to a local provider for follow-up helps to ensure everyone requiring follow-up can access care. Individuals testing positive for HPV 16 or 18 are referred directly to colposcopy [36]. British Columbia launched a province-wide self-collection program in January 2024.

### 7.7. Prince Edward Island’s Self-Collection Experience

Prince Edward Island ran a three-month pilot self-collection program over the summer of 2024 in various clinical settings, including traditional cervical cancer screening clinics, primary care clinics, indigenous community clinics, and a sexual health clinic. Patients were given the self-collection kits and educational materials in clinics and asked to do the testing while on site. Approximately 400 tests were collected. Patients were easy to recruit and reported being comfortable with the test. A key element in the readiness to run the pilot program was advocacy and a change in legislation to allow nurses to follow up and refer to colposcopy. Prince Edward Island has the highest rate of unattached patients in Canada, up to 40%, including many new immigrants. Without the ability of nurses to refer individuals who require colposcopy, most of these people would be at risk of being lost to follow up [39].

They identified that significant education is required for providers and patients. Providers had challenges understanding inclusion/exclusion criteria and would prefer very clear national standardization. Sustainability is very important; the pilot process would not be sustainable throughout the province as piloted without the oversight of the cervical screening program to ensure appropriate follow up.

### 7.8. Learning from Other Countries

An early modeling study out of the Netherlands [37] suggests that if a highly sensitive HPV test is used and uptake increases, especially among those at highest risk of developing cervical cancer, self-collection will always lead to a net saving of life years and quality-adjusted life years at the population level, even if all people are screened with self-sampling and nobody opts for provider-sampling. There is some concern about the impact of self-collection for HPV testing. A slight reduction in test sensitivity with self-collected samples, combined with growing preference for self-collection but insufficient uptake among high-risk groups, may reduce or even eliminate its overall benefit at the population level.

However, information from Australia rebuts the worry of reduced sensitivity based on their HPV self-collection experience. Initial validation studies of the HPV self-collection swab with clinician-taken swabs showed a good concordance and sensitivity between the samples [38]. The Australian National Cervical Screening Program (NCSP) then completed a real-world study comparing HPV self-collected swabs to clinician-collected swabs in the under- or never-screened population from January to June 2022. Results showed an equivalent HPV detection rate [40]. The NCSP expanded the program to offer HPV self-collection to the entire population of Australia in mid 2022, and as of the first quarter of 2025, 40% of their HPV tests are self-collected [40].

### 7.9. Self-Collection for Those at Highest Risk of Cervical Cancer

HPV self-collection is most beneficial to populations that are at risk of non-screening, and has the potential to improve equity in cervical screening participation. These include remote/rural populations, indigenous and northern populations, newly immigrated Canadians, 2SLGBTQIA+ individuals, and any marginalized population. Equity does not mean everyone doing the same thing everywhere-it means every setting receives the most appropriate resources for their situation, which may entail different tools and steps. Identifying why people choose not to be screened is important and challenging.

Distribution of cervical screening self-collection kits may be performed through mail outs, pick up at provider offices, electronic invitations and reminders, and by offering more convenient screening locations and times [35].

### 7.10. HPV Testing Education

Education is required for healthcare providers and the public who require HPV testing. A national survey of 3724 screening-eligible Canadians (which included 1853 under-screened women, defined as women who had never been screened or had been screened in over 3 years) examined women’s knowledge, attitudes, and preferences regarding the transition from Pap to HPV testing [41]. Findings showed that personal barriers reduced the likelihood of HPV testing, while confidence in HPV-based screening and a sense of autonomy increased intentions to participate across both adequately screened and under-screened groups. Attitudinal factors (beliefs and perceived barriers) were stronger predictors of screening acceptability than sociodemographics or knowledge, which showed mixed effects. It is essential that Canadians understand the evidence supporting later initiation and longer intervals with HPV screening. Empowering messages that emphasize autonomy and confidence, such as “cervical screening is in your hands” and “simple, safe, and can be done at home”, are recommended. Additionally, most women preferred to receive information about cervical cancer and screening from provincial public health authorities, followed by HCPs. Also, most preferred to receive invitations, reminders, and results by email. This suggests an important need for technology to be linked to HPV screening implementation delivery. Lastly, one practical issue is that many Canadians do not have a family doctor. A total of 35% of women who were under screened did not have access to a family provider, whereas 16% did not have access to a family provider in the adequately screened group.

Increasing awareness and decreasing barriers in under-screened populations may be improved using targeted and culturally relevant communication approaches created in partnership with healthcare professionals and communities [35].

Consider partnering with organizations that serve underserved and vulnerable populations, with education appropriate to specific groups, e.g., culture, education level, age, language, format, and media, etc.

### 7.11. Centralized Collection of Data

High-quality, comprehensive, population-based registries are necessary to track and analyze data on HPV immunization, cervical screening, and HPV-related cancers within a population. Registries serve several purposes, including the following:

- Assess vaccination uptake and trends over time. This can help target interventions to improve vaccination rates in defined populations.
- Assess cervical screening uptake and demographics of those participating, including identifying those who have never been screened or those overdue for screening. These individuals can be targeted in campaigns to improve screening, such as screening invitations, recall letters, social media, and community outreach through trusted providers and leaders.
- Accurately monitor the success of immunization and screening programs based on clinical outcomes. Where are we seeing successes (e.g., less HPV-related disease), and where do we need to focus efforts to see an overall population benefit?
- Predict future needs and address these in a data-informed manner.

Some provinces have registries in place to track vaccination and screening, while others do not. Registries serve a valuable function as they can help us identify our population who needs further attention to increase their screening and vaccination rates.

### 7.12. Follow up for People Who Test Positive for HPV

Colposcopy, a clinical examination of the cervix, vagina, and vulva using a magnifying lens, is the standard follow up to a positive cervical screening test, including a positive HPV test. Access to colposcopy and willingness to follow up are critical for reducing cancer incidence. Each province and territory has its own criteria for referral to colposcopy services [42], and locations of clinics as well as wait times vary across the country.

Currently there is no standardized approach to collecting and monitoring data on follow-up care in Canada. Underserved jurisdictions are especially vulnerable to lack of follow up. Sharing lessons learned from jurisdictions that have addressed the needs of underserved populations may help to address equitable access to follow-up care.

## 8. Recommendations: Screening

1. Gather and share experience and expertise developed in different jurisdictions.
2. Develop and evaluate a comprehensive, multi-faceted approach that includes strategies tailored to Canada’s many different populations: new Canadians; Inuit, Métis, and First Nations; northern, remote, and rural populations; lower income neighborhoods; 2SLGBTQIA+ individuals; people of certain races and ethnicities (visible minorities, Black); and others.
3. Implement HPV testing that includes access to HPV self-collection and evaluate targeted and culturally relevant education, information, and awareness-raising initiatives on the importance of screening and follow up. There should be a focus on under-screened populations, as access to HPV self-collection can reduce barriers to screening, particularly in marginalized populations.
4. Identify, develop, implement, and evaluate targeted strategies to address inequities and improve the accessibility of cervical cancer screening, such as HPV self-collection. Support development of provincial registries to more effectively identify un/underscreened individuals and allow for targeted education and tactics to encourage screening. Registries can also improve, and support follow up of abnormal results and support referral to colposcopy.
5. Support and encourage partnerships, collaborations, experience, and resource sharing to overcome barriers.
6. Support healthcare professionals to increase participation in cervical cancer screening.

HPV testing finds more pre-cancers.

Precancer treatment saves lives and is efficient.

## Figures and Tables

**Table 2:** NACI updated guidance on HPV vaccines [25].

Population	9vHPV * Vaccine
Immunocompetent individuals aged 9 to 20 years old	Single dose
Immunocompetent individuals aged 27 and older	Two doses, administered at least 24 weeks apart
Individuals considered immunocompromised, individuals living with HIV	Three-dose schedule
Pregnant individuals +	No evidence to date of increased risk of adverse pregnancy or fetal outcomes associated with HPV vaccination during pregnancy. However, HPV vaccination is not recommended during pregnancy and should wait until the pregnancy is complete.
Equity-denied groups, including First Nations, Inuit and Métis people, some of whom face disproportionately high rates of HPV-associated cancers and lower rates of HPV immunization	NACI recommends dedicated efforts to improve HPV vaccination coverage

* NACI recommends the use of the 9vHPV vaccine to provide protection from the greatest number of vaccine-preventable strains. + HPV vaccination can be given while breastfeeding.

**Table 3:** Cancers that result from HPV infections [2,5,11,14,31].

Type of Cancer	% Resulting from HPV
Cervical cancer	90%
Anal cancers	80–90%
Vaginal cancer	65%
Mouth and throat cancers	45–95%
Penile cancers	40–50%
Vulvar cancers	40%

## Abbreviations

The following abbreviations are used in this manuscript:

NACI	National Advisory Committee on Immunization
HPV	Human papillomavirus
HSIL	High-grade squamous intraepithelial lesion
PHAC	Public Health Agency of Canada
2SLGBTQIA+	Two-Spirit, Lesbian, Gay, Bisexual, Transgender, Queer or Questioning, Intersex, Asexual, and other sexual and gender diverse communities

## Appendix A. Advisory Committee Members

**Chair**
**Shannon Salvador**, MD, MSc, FRCSC
   President, Society of Gynecologic Oncology of Canada
   Associate Professor, McGill University
   Gynecology Oncology, Jewish General Hospital
   Montreal, QC
**Members**
**James Bentley**, MBChB, FRCSC
   Professor, Division of Gynaecologic Oncology, Department Head, Obstetrics and Gynecology, Dalhousie University
   Halifax, NS
**Vivien Brown**, MDCM, CCFP, FCFP, MSCP
   Family Physician
   Assistant Professor, Department of Family and Community Medicine, University of Toronto
   Chair, HPV Task Force at the Federation of Medical Women in Canada
   Toronto, ON
**Monique A. Bertrand**, MD, FRCSC
   Professor Emeritus, Department of Obstetrics and Gynecology,
   Western University,
   London, Ontario
**Céline Bouchard**, MD, FRCSC
  Obstetrician-gynecologist, director of Clinique de Recherche en Santé de la Femme
   Quebec, QC
**Jennifer Brown Broderick**, MD, MSc, FRCSC, DABOG
   Assistant Professor Department of Oncology, University of Saskatchewan
   Saskatchewan Cancer Agency
   Saskatoon, SK
**Catriona Buick**, RN, PhD, CON(C)
   President, Canadian Association of Nurses in Oncology
   Assistant Professor, York University
   Oncology Nurse Clinician Scientist, Odette Cancer Centre
   Affiliate Scientist, Sunnybrook Research Institute
   Toronto, ON
**Krista Cassell**, MD, FRCSC
   Gynecologist, Queen Elizabeth Hospital, Charlottetown
   Associate Professor, Department of Obstetrics and Gynecology, Dalhousie University
   Charlottetown, PE
**Genevieve Chaput**, MD
   Attending Physician, McGill University Health Centre
   Chair, Cancer Care Members Interest Group (MIG), College of Family Physicians of Canada
**Kelly Cull**
   Director, Advocacy, Canadian Cancer Society
   Halifax, NS
**Sheri Fitzpatrick-Poulain**
   HPV Portfolio Lead, Federation of Medical Women of Canada
   Vancouver, BC
**Diane Francoeur**, MD, FRCSC
   Associate Clinical Professor, Faculty of Medicine, Department of Obstetrics and Gynecology, University of Montreal
   Sainte-Justine, QC
**Manjula Ganesh**
   Patient Partner
   Toronto, Ontario
**Ronald Grimshaw**, MD, FRCSC
   Post-Retirement Associate Professor, Department of Obstetrics and Gynecology, Dalhousie University
   Halifax, NS
**Dave Hawkes**, PhD
   Director Australian HPV Reference Laboratory,
   Australian Centre for the Prevention of Cervical Cancer
   Melbourne, Australia
**Sarah Kean**, MD, FRCSC
   Assistant Professor, Division of Gynecologic Oncology, Department of Obstetrics, Gynecology, and Reproductive Sciences, College of Medicine, Rady Faculty of Health Sciences, University of Manitoba,
   Winnipeg, MB
**Eileen Kilfoil**, MITE
   Manager, Cancer Screening Programs, Nova Scotia Cancer Care Program
   Halifax, NS
**Roni Kraut** CPA, MD, CCFP, MSc
   Family Physician
   Assistant Professor, Department of Family Medicine, University of Alberta
   Edmonton, AB
**Amélie McFadyen**, MA
   Chief Executive Officer, HPV Global Action
   Montreal QC
**Meg McLachlin**, MD, FRCPC
   Professor Emeritus, Pathology
   Western University
   London, ON
**Marie-Helene Mayrand**, MD, PhD, FRCSC
   Gynecologist, Department of obstetrics-gynecology, Centre hospitalier de l’Université de Montréal
   Professor, Department of obstetrics-gynecology, Université de Montréal
   Montreal, QC
**Lananh Nguyen**, MSc, MD
   Canadian Representative, ACCESS International Consensus Group on Cervical Cancer
   Associate Professor, Department of Laboratory Medicine and Pathobiology, University of Toronto
   Laboratory Medicine and Pathology, Unity Health Toronto
   Toronto, Ontario
**Se-Inyenede Onobrakpor**, MD, MPH, MHM, CHE
   Program Manager, Alberta Cervical Cancer Screening Program
   Calgary, AB
**Elizabeth Pavlovic**, BN, RN, MN, NP
   Assistant Teaching Professor, Faculty of Nursing, University of New Brunswick
   Moncton, NB
**Samara Perez**, PhD
   Psychologist and Clinician-Scientist, Cedars Cancer Centre McGill University Health Centre
   Assistant professor, Department of Oncology, McGill University
**Catherine Popadiuk** MD, FRCSC
   Medical Director Cervical Screening Initiatives Newfoundland
   Associate Professor, CPD Lead, Obstetrics and Gynecology, Memorial University
   St John’s, NL
**Lily Proctor**, MD, FRCSC
   Gynecologic oncologist, BC Cancer Agency and Vancouver General Hospital
   Medical Director, Cervical Cancer Screening Program, BC Cancer
   Vancouver, BC
**Brandon Purcell**
   Advocacy Manager, Canadian Cancer Society
   Ottawa, ON
**Carla Roberts**, MD, FRCSC
   Assistant Professor, Department of Obstetrics and Gynecology Dalhousie University
   Assistant Professor, Memorial University
   Moncton, NB
**Gilla K. Shapiro**, MPA/MPP, PhD, CPsych
   Psychologist and Clinician-Scientist, Department of Supportive Care, Princess Margaret Cancer Centre
   Assistant Professor, Department of Psychiatry and Dalla Lana School of Public Health, University of Toronto
   Toronto, ON
**Chantal Sauvageau**, MD, MSc. FRCPC
   Quebec Public Health Institute
   Professor, Department of Social and Preventive Medicine, Faculty of Medicine, Laval University
   Researcher, CHU de Quebec-University Laval Research Center
   Quebec, QC
**Joanne Sivertson**, MD, FRCSC
   Provincial Department Head Obstetrics and Gynecology, Saskatchewan Health Authority and University of Saskatchewan
   Medical Advisor, Saskatchewan Cervical Cancer Screening Program, Saskatchewan Cancer Agency
   Prince Albert, SK
**Cidalia Sluce**
   National Manager, Society of Gynecologic Oncology of Canada
   Winnipeg, MB
**Helena Sonea**
   Director, Advocacy, Canadian Cancer Society
   Ottawa, ON
**Anna N. Wilkinson,** MSc., MD, CCFP, FCFP
   Associate Professor, University of Ottawa, Department of Family Medicine
   Family Physician, The Ottawa Academic Family Health Team
   GP Oncologist, The Ottawa Hospital Cancer Centre
   Program Director, PGY-3 FP-Oncology
**Karla Willows**, MD, MSc, FRCSC
   President, Canadian Colposcopy Society
   Assistant Professor, Dalhousie University
   Gynecologic Oncologist, Nova Scotia Health
   Halifax, NS
**Carmen Wyton**
   President, Women’s Health Coalition of Canada
   St Albert, AB
**Adrienne Zuck**, RN
   Four Directions Community Health Centre
   Regina, SK
